# Supplementary material for: Programmable conjugative CRISPR interference targeting genotoxin in the gut
Source: Res Sq. 2025 Oct 14:rs.3.rs-7761902. Preprint. [Version 1] doi: 10.21203/rs.3.rs-7761902/v1 (PMC12633163; doi:10.21203/rs.3.rs-7761902/v1)
Supplement: Supplement 1 [file NIHPPrs7761902v1-supplement-1.pdf]

Table S1, Alignment of insertional sequences discovered within the open reading frame of the Cas9 gene.

```
CLUSTAL O(1.2.4) multiple sequence alignment


B73_3           GGCAAAGCACTGAGAGATCCCCTCATAATTTCCCCAAAGCGTA---ACCATGTGT----G   53
B4387_1         TGCTAAGTCCTGAGAGATCCCCTCATAATTTCCCCAAAACGTA---ACCATGTGT----G   53
B4387_2         TGCTAAGTCCTGAGAGATCCCCTCATAATTTCCCCAAAACGTA---ACCATGTGT----G   53
A287_1          TACTCAATACTGATGAATCCCCTAATGATTTTTATCAAAATCATTAAGTTAAGGTAGATA   60
B73_1           TGCTAAGTCCTGATGAATCCCCTAATGATTTTTATCAAAATCATTAAGTTAAGGTAGATA   60
B73_2           TGCTAAGTCCTGATGAATCCCCTAATGATTTTTATCAAAATCATTAAGTTAAGGTAGATA   60
B4387_3         -CAAATAGCGCTACTAATCCCCTAATGATTTTGGTAAAAATCATTAAGTTAAGGTGGATA   59
                            *   ******* ** ****     **    *   *      **

B73_3           AATAAATTTTGAGCTAGT---AGGGTT-----------------GCAGCCACGAGTAAGT   93
B4387_1         AATAGATTTTGAGTAAGC---AGGGTT-----------------GCAGCCACGAGTGAGT   93
B4387_2         AATAGATTTTGAGTAAGC---AGGGTT-----------------GCAGCCACGAGTGAGT   93
A287_1          CACATCTTGTCATATGATCAAATGGTTTCGCCAAAAATCAATAATCAGACAACAAAATGT   120
B73_1           CACATCTTGTCATATGATCAAATGGTTTCGCCAAAAATCAATAATCAGACAACAAAATGT   120
B73_2           CACATCTTGTCATATGATCAAATGGTTTCGCCAAAAATCAATAATCAGACAACAAAATGT   120
B4387_3         CACATCTTGTCATATGATCAAATGGTTTCGCGAAAAATCAATAATCAGACAACAAGATGT   119
                 * *  ** * *         * ****                  *** **  *    **

B73_3           CTTCCCTTGTTATTG------------TGTAGCCAGAATGCCGCAAAACTTCCATGCCTA   141
B4387_1         CTTCCCTTGTTATTG------------TGTAGCCAGAATGCCGCAAAACTTCCATGCCTA   141
B4387_2         CTTCCCTTGTTATTG------------TGTAGCCAGAATGCCGCAAAACTTCCATGCCTA   141
A287_1          GCGAACTCGATATTTTACACGACTCTCTTTACCAATTCTGCCCCGAATTA--C--ACTTA   176
B73_1           GCGAACTCGATATTTTACACGACTCTCTTTACCAATTCTGCCCCGAATTA--C--ACTTA   176
B73_2           GCGAACTCGATATTTTACACGACTCTCTTTACCAATTCTGCCCCGAATTA--C--ACTTA   176
B4387_3         GCGAACTCGATATTTTACACGACTCTCTTTACCAATTCTGCCCCGAATTA--C--ACTTA   175
                     ** * ****             * ** * *   **** * **     *   * **

B73_3           AGCGAACTGTTGAGAGTACGTTTCGATTTC-TGACTGTGTTAGCCTGGAAGTGCTTGTCC   200
B4387_1         AGCGAACTGTTGAGAGTACGTTTCGATTTC-TGACTGTGTTAGCCTGGAAGTGCTTGTCC   200
B4387_2         AGCGAACTGTTGAGAGTACGTTTCGATTTC-TGACTGTGTTAGCCTGGAAGTGCTTGTCC   200
A287_1          AAACGACTCAACAGCTTAACGTTGGCTTGCCACGCCTTACTTGACTGTAAAA----CTCT   232
B73_1           AAACGACTCAACAGCTTAACGTTGGCTTGCCACGCCTTACTTGACTGTAAAA----CTCT   232
B73_2           AAACGACTCAACAGCTTAACGTTGGCTTGCCACGCCTTACTTGACTGTAAAA----CTCT   232
B4387_3         AAACGACTCAACAGCTTAACGTTGGCTTGCCACGCATTACTTGACTGTAAAA----CTCT   231
                *    ***    **  **   ** * ** *    *  *  * * *** **       **

B73_3           CAACCTTGTTTCTGAGCATGAACGCCCGCAAGCCAACATGTTAGTTGAAGCATCAGGGCG   260
B4387_1         CAACCTTGTTTCTGAGCATGAACGCCCGCAAGCCAACATGTTAGTTGAAGCATCAGGGCG   260
B4387_2         CAACCTTGTTTCTGAGCATGAACGCCCGCAAGCCAACATGTTAGTTGAAGCATCAGGGCG   260
A287_1          CAC---TCTTACCGAACTTGGCC----GTAACCTGCCA------------ACCAAAGCG   272
B73_1           CAC---TCTTACCGAACTTGGCC----GTAACCTGCCA------------ACCAAAGCG   272
B73_2           CAC---TCTTACCGAACTTGGCC----GTAACCTGCCA------------ACCAAAGCG   272
B4387_3         CAC---TCTTACCGAACTTGGCC----GTAACCTGCCA------------ACCAAAGCG   271
                **    * ** * ** * **  *    * ** *   **             * **  ***
```

```
B73_3           ATT-AGCAGCATGATATCAAAACGCTCTGAGCTGCTCGTTCGGCTATGGCGTAGGCCTAG   319
B4387_1         ATT-AGCAGCATGATATCAAAACGCTCTGAGCTGCTCGTTCGGCTATGGCGTAGGCCTAG   319
B4387_2         ATT-AGCAGCATGATATCAAAACGCTCTGAGCTGCTCGTTCGGCTATGGCGTAGGCCTAG   319
A287_1          AGAACAAAACATAACATCAAACGA--ATCGACCGATTGTTAGGTAATCGTCACCTCCACA   330
B73_1           AGAACAAAACATAACATCAAACGA--ATCGACCGATTGTTAGGTAATCGTCACCTCCACA   330
B73_2           AGAACAAAACATAACATCAAACGA--ATCGACCGATTGTTAGGTAATCGTCACCTCCACA   330
B4387_3         AGAACAAAACATAACATCAAACGA--ATCGACCGATTGTTAGGTAATCGTCACCTCCACA   329
                *      * *** * ******      *   * * * *** **  ** *      **

B73_3           TCCGTAGGCAGGACTTTTCAAGTCTCGGAAGGTTTCTTCAATCTGC--ATTCGCTTCGAA   377
B4387_1         TCCGTAGGCAGGACTTTTCAAGTCTCGGAAGGTTTCTTCAATCTGC--ATTCGCTTCGAA   377
B4387_2         TCCGTAGGCAGGACTTTTCAAGTCTCGGAAGGTTTCTTCAATCTGC--ATTCGCTTCGAA   377
A287_1          AAGAGCGACT-CGC-TGTATACCGTTGGCATGCTAGCTTTATCTGTTCGGGCAATACGAT   388
B73_1           AAGAGCGACT-CGC-TGTATACCGTTGGCATGCTAGCTTTATCTGTTCGGGCAATACGAT   388
B73_2           AAGAGCGACT-CGC-TGTATACCGTTGGCATGCTAGCTTTATCTGTTCGGGCAATACGAT   388
B4387_3         AAGAGCGACT-CGC-TGTATACCGTTGGCATGCTAGCTTTATCTGTTCGGGCAATACGAT   387
                      * *    * * *  *   * ** * * *   *  *****      *  * ***

B73_3           TAGATATTAACAAGTTGTTTGGGTGTTCGAATTTCAACAGGTAAGTTAGTTGCTAGAACC   437
B4387_1         TAGATATTAACAAGTTGTTTGGGTGTTCGAATTTCAACAGGTAAGTTAGTTGCTAGAATC   437
B4387_2         TAGATATTAACAAGTTGTTTGGGTGTTCGAATTTCAACAGGTAAGTTAGTTGCTAGAATC   437
A287_1          GCCCATTGTACTTGTTGAC---TGGTCTGATATCCGTGAGCAAAAACGGCTTATGGTATT   445
B73_1           GCCCATTGTACTTGTTGAC---TGGTCTGATATCCGTGAGCAAAAACGGCTTATGGTATT   445
B73_2           GCCCATTGTACTTGTTGAC---TGGTCTGATATCCGTGAGCAAAAACGGCTTATGGTATT   445
B4387_3         GCCCATTGTACTTGTTGAC---TGGTCTGATATTCGTGAGCAAAAACGACTTATGGTATT   444
                      *  **  ****       **  **  * *   **  **      *  * * *

B73_3           CATGGCTCCTTTGCCGACGCTGAGTAGATTTTAGGTGACGGGTGGTGACAATGAGTCCGT   497
B4387_1         CATGGCTCCTTTGCCGACGCTGAGTAGATTTTAGGTGACGGGTGGTGACAATGAGTCCGT   497
B4387_2         CATGGCTCCTTTGCCGACGCTGAGTAGATTTTAGGTGACGGGTGGTGACAATGAGTCCGT   497
A287_1          GCGAGCTTCAG---TCGCACTACACGGTCGTTCTGTTACTCTTTATGAGAAAGCGTTCCC   502
B73_1           GCGAGCTTCAG---TCGCACTACACGGTCGTTCTGTTACTCTTTATGAGAAAGCGTTCCC   502
B73_2           GCGAGCTTCAG---TCGCACTACACGGTCGTTCTGTTACTCTTTATGAGAAAGCGTTCCC   502
B4387_3         GCGAGCTTCAG---TCGCACTACACGGTCGTTCTGTTACTCTTTATGAGAAAGCGTTCCC   501
                    *** *        * **     *   **  ** **   *  *** ** * ** *

B73_3           G--TCGAGCGCTGATTTTT--------------------TCGGCCTTTAGAGCGAGATT   534
B4387_1         G--TCGAGCGCTGATTTTT--------------------TCGGCCTTTAGAGCGAGATT   534
B4387_2         G--TCGAGCGCTGATTTTT--------------------TCGGCCTTTAGAGCGAGATT   534
A287_1          GCTTTCAGAGCAATGTTCAAAGAAAGCTCATGACCAATTTCTAGCCGACCTTGCGAGCAT   562
B73_1           GCTTTCAGAGCAATGTTCAAAGAAAGCTCATGACCAATTTCTAGCCGACCTTGCGAGCAT   562
B73_2           GCTTTCAGAGCAATGTTCAAAGAAAGCTCATGACCAATTTCTAGCCGACCTTGCGAGCAT   562
B4387_3         GCTTTCAGAGCAATGTTCAAAGAAAGCTCATGACCAATTTCTAGCCGACCTTGCGAGCAT   561
                *  *  ** **    **                          ***      *****  *
```

```
B73_3           TATACAATAGA----------ATTTGGCATGAGATTGGATTGCTTTTAGTCAGCCTCTTA   584
B4387_1         TATACAATAGA----------ATTTGGCATGAGATTGGGTTGCTTTTAGTCAGCCTCTTA   584
B4387_2         TATACAATAGA----------ATTTGGCATGAGATTGGGTTGCTTTTAGTCAGCCTCTTA   584
A287_1          TCTACCGAGTAACACCACACCGCTCATTGTCAGTGATGCTGGCTTTAAAGT-----GCCA   617
B73_1           TCTACCGAGTAACACCACACCGCTCATTGTCAGTGATGCTGGCTTTAAAGT-----GCCA   617
B73_2           TCTACCGAGTAACACCACACCGCTCATTGTCAGTGATGCTGGCTTTAAAGT-----GCCA   617
B4387_3         TCTACCGAGTAACACCACACCGCTCATTGTCAGTGATGCTGGCTTTAAAGT-----GCCA   616
                * ***     *            *     * **    * * ***** *           *

B73_3           TAGCCTAAAGTCTTTGAGTGACTAGATGACATATCATGTAAGT---TGCTGATAGGTTTC   641
B4387_1         TAGCCTAAAGTCTTTGAGTGACTAGATGACATATCATGTAAGT---TGCTGATAGGTTTC   641
B4387_2         TAGCCTAAAGTCTTTGAGTGACTAGATGACATATCATGTAAGT---TGCTGATAGGTTTC   641
A287_1          TGGTATAAATCCGTTGAGAAGCTGGGTTGGTACTGGTTAAGTCGAGTAAGAGGAAAAGTA   677
B73_1           TGGTATAAATCCGTTGAGAAGCTGGGTTGGTACTGGTTAAGTCGAGTAAGAGGAAAAGTA   677
B73_2           TGGTATAAATCCGTTGAGAAGCTGGGTTGGTACTGGTTAAGTCGAGTAAGAGGAAAAGTA   677
B4387_3         TGGTATAAATCCGTTGAGAAGCTGGGTTGGTACTGGTTAAGTCGAGTAAGAGGAAAAGTA   676
                * *  ****  * *****   ** * *      *  *  *      *      *    *

B73_3           CAGTTTTCCGCTCCTAGGTCTGCATATTGTACTT-----TTCCTCTTACTCGACTTAACC   696
B4387_1         CAGTTTTCCGCTCCTAGGTCTGCATATTGTACTT-----TTCCTCTTACTCGACTTAACC   696
B4387_2         CAGTTTTCCGCTCCTAGGTCTGCATATTGTACTT-----TTCCTCTTACTCGACTTAACC   696
A287_1          CAATATGCAGACCTAGGAGCGGAAAACTGGAAACCTATCAGCAACTTACATGATATGTCA   737
B73_1           CAATATGCAGACCTAGGAGCGGAAAACTGGAAACCTATCAGCAACTTACATGATATGTCA   737
B73_2           CAATATGCAGACCTAGGAGCGGAAAACTGGAAACCTATCAGCAACTTACATGATATGTCA   737
B4387_3         CAATATGCAGACCTAGGAGCGGAAAACTGGAAACCTATCAGCAACTTACATGATATGTCA   736
                ** * * * *  *   *  * * * * ** *          *  *****  **  *  *

B73_3           AGTACCAACCCAGCTTCTCAACGGATTTATACCATGGCACTTTAAAGCCAGCATCACTGA   756
B4387_1         AGTACCAACCCAGCTTCTCAACGGATTTATACCATGGCACTTTAAAGCCAGCATCACTGA   756
B4387_2         AGTACCAACCCAGCTTCTCAACGGATTTATACCATGGCACTTTAAAGCCAGCATCACTGA   756
A287_1          --------TCTAGTCACTCAAAGACTTTAGGCTATAAGAGGC---------------TGA   774
B73_1           --------TCTAGTCACTCAAAGACTTTAGGCTATAAGAGGC---------------TGA   774
B73_2           --------TCTAGTCACTCAAAGACTTTAGGCTATAAGAGGC---------------TGA   774
B4387_3         --------TCTAGTCACTCAAAGACTTTAGGCTATAAGAGGC---------------TGA   773
                         * **   ***** *  ****  * **   *                  ***

B73_3           CAATGAGCGGTGTGGTGTTACTCGGTAGAATGCTCGCAAGGTCGGCTAGAAATTGGTCAT   816
B4387_1         CAATGAGCGGTGTGGTGTTACTCGGTAGAATGCTCGCAAGGTCGGCTAGAAATTGGTCAT   816
B4387_2         CAATGAGCGGTGTGGTGTTACTCGGTAGAATGCTCGCAAGGTCGGCTAGAAATTGGTCAT   816
A287_1          CTAAAAG------------------------CAACCCAATCTC---------ATGCCAAA   801
B73_1           CTAAAAG------------------------CAACCCAATCTC---------ATGCCAAA   801
B73_2           CTAAAAG------------------------CAACCCAATCTC---------ATGCCAAA   801
B4387_3         CTAAAAG------------------------CAATCCAATCTC---------ATGCCAAA   800
                * *  **                             ***  **          **   *
```

```
B73_3          GAGCTTTCTTTGAACATTGCTCTGAAAGCGGGAAC--------------------GCTTT   856
B4387_1        GAGCTTTCTTTGAACATTGCTCTGAAAGCGGGAAC--------------------GCTTT   856
B4387_2        GAGCTTTCTTTGAACATTGCTCTGAAAGCGGGAAC--------------------GCTTT   856
A287_1         TTCTATTGTATAAATCTCGCTCTAAAGGCCGAAAAAATCAGCGCTCGACACGGACTCATT   861
B73_1          TTCTATTGTATAAATCTCGCTCTAAAGGCCGAAAAAATCAGCGCTCGACACGGACTCATT   861
B73_2          TTCTATTGTATAAATCTCGCTCTAAAGGCCGAAAAAATCAGCGCTCGACACGGACTCATT   861
B4387_3        TTCTATTGTATAAATCTCGCTCTAAAGGCCGAAAAAATCAGCGCTCGACACGGACTCATT   860
                    ** * * **  * ***** ** ** * **                      * **

B73_3          CTCATAAAGAGTAACAGAACGACCGTGTAGT---GCGACTGAAGCTCGCAATACCATAAG   913
B4387_1        CTCATAAAGAGTAACAGAACGACCGTGTAGT---GCGACTGAAGCTCGCAATACCATAAG   913
B4387_2        CTCATAAAGAGTAACAGAACGACCGTGTAGT---GCGACTGAAGCTCGCAATACCATAAG   913
A287_1         GTCACCACCCGTCACCTAAAATCTACTCAGCGTCGGCAAAGGAGCCATGGATTCTAGCAA   921
B73_1          GTCACCACCCGTCACCTAAAATCTACTCAGCGTCGGCAAAGGAGCCATGGATTCTAGCAA   921
B73_2          GTCACCACCCGTCACCTAAAATCTACTCAGCGTCGGCAAAGGAGCCATGGATTCTAGCAA   921
B4387_3        GTCACCACCCGTCACCTAAAATCTACTCAGCGTCGGCAAAGGAGCCATGGGTTCTAGCAA   920
                ***  *   ** **  **   *     **    *  *  * ***      * * *  *

B73_3          TCGTTTTTGCTCACGAATATCAGACCAGTCAAC---AAGTACAATGGGCATCGTATTGCC   970
B4387_1        CCGTTTTTGCTCACGGATATCAGACCAGTCAAC---AAGTACAATGGGCATCGTATTGCC   970
B4387_2        CCGTTTTTGCTCACGGATATCAGACCAGTCAAC---AAGTACAATGGGCATCGTATTGCC   970
A287_1         CTAACTTACCTGTTGAAATTCGAACACCCAAACAACTTGTTAATATCTATTCGAAGC--G   979
B73_1          CTAACTTACCTGTTGAAATTCGAACACCCAAACAACTTGTTAATATCTATTCGAAGC--G   979
B73_2          CTAACTTACCTGTTGAAATTCGAACACCCAAACAACTTGTTAATATCTATTCGAAGC--G   979
B4387_3        CTAACTTACCTGTTGAAATTCGAACACCCAAACAACTTGTTAATATCTATTCGAAGC--G   978
                    **  **   *  *  **  **     ***     **  *       *** *

B73_3          CGAACAGATAAAGCTAGCATGCCAACGGTATACAGCGAGTCGCT-CTTTGTG-GAGGTGA   1028
B4387_1        CGAACAGATAAAGCTAGCATGCCAACGGTATACAGCGAGTCGCT-CTTTGTG-GAGGTGA   1028
B4387_2        CGAACAGATAAAGCTAGCATGCCAACGGTATACAGCGAGTCGCT-CTTTGTG-GAGGTGA   1028
A287_1         AATGCAGATTGAAGAAACCTTCCGAGACTTGAAAAGTCCTGCCTACGGACTAGGCCTACG   1039
B73_1          AATGCAGATTGAAGAAACCTTCCGAGACTTGAAAAGTCCTGCCTACGGACTAGGCCTACG   1039
B73_2          AATGCAGATTGAAGAAACCTTCCGAGACTTGAAAAGTCCTGCCTACGGACTAGGCCTACG   1039
B4387_3        AATGCAGATTGAAGAAACCTTCCGAGACTTGAAAAGTCCTGCCTACGGACTAGGCCTACG   1038
                   *****  *   * * * ** *   *  * *     *  ** *    *  *

B73_3          CGATTACCTAACAATCG-GTCGATTCGT-TTGATGTTATGTTTTGTTCTCGCTTTGG---   1083
B4387_1        CGATTACCTAACAATCG-GTCGATTCGT-TTGATGTTATGTTTTGTTCTCGCTTTGG---   1083
B4387_2        CGATTACCTAACAATCG-GTCGATTCGT-TTGATGTTATGTTTTGTTCTCGCTTTGG---   1083
A287_1         CCATAGCCGAACGAGCAGCTCAGAGCGTTTTGATATCATGC-TGCTAATCGCCCTGATGC   1098
B73_1          CCATAGCCGAACGAGCAGCTCAGAGCGTTTTGATATCATGC-TGCTAATCGCCCTGATGC   1098
B73_2          CCATAGCCGAACGAGCAGCTCAGAGCGTTTTGATATCATGC-TGCTAATCGCCCTGATGC   1098
B4387_3        CCATAGCCGAACGAGCAGCTCAGAGCGTTTTGATATCATGC-TGCTAATCGCCCTGATGC   1097
               * **  ** *** * *   **    *** ***** * ***  *  *  ****  **
```

```
B73_3           ----------TTGGCAGGTTACGGCCAAGTTCGGTAAGAGTGAGAGTTTTACAGTCAAGT   1133
B4387_1         ----------TTGGCAGGTTACGGCCAAGTTCGGTAAGAGTGAGAGTTTTACAGTCAAGT   1133
B4387_2         ----------TTGGCAGGTTACGGCCAAGTTCGGTAAGAGTGAGAGTTTTACAGTCAAGT   1133
A287_1          TTCAACTAACATGTTGGCTTGCGGGCGTTCATGCTCAGAAACAAGGTTG---GGACAAGC   1155
B73_1           TTCAACTAACATGTTGGCTTGCGGGCGTTCATGCTCAGAAACAAGGTTG---GGACAAGC   1155
B73_2           TTCAACTAACATGTTGGCTTGCGGGCGTTCATGCTCAGAAACAAGGTTG---GGACAAGC   1155
B4387_3         TTCAACTAACATGTTGGCTTGCGGGCGTTCATGCTCAGAAACAAGGTTG---GGACAAGC   1154
                           **   * ** *** *      * * ***   *  ***     * ****

B73_3           AATGCGTGGCAAGCCAACGTTAAGCTGTTGAGTCGTTTTAAGTGTAATTCG-----GGGC   1188
B4387_1         AAGGCGTGGCAAGCCAACGTTAAGCTGTTGAGTCGTTTTAAGTGTAATTCG-----GGGC   1188
B4387_2         AAGGCGTGGCAAGCCAACGTTAAGCTGTTGAGTCGTTTTAAGTGTAATTCG-----GGGC   1188
A287_1          ACTTCCAGGCTAACACA--GTCAGAAATCGAAACGTACTCTCAACAGTTCGCTTAGGCAT   1213
B73_1           ACTTCCAGGCTAACACA--GTCAGAAATCGAAACGTACTCTCAACAGTTCGCTTAGGCAT   1213
B73_2           ACTTCCAGGCTAACACA--GTCAGAAATCGAAACGTACTCTCAACAGTTCGCTTAGGCAT   1213
B4387_3         ACTTCCAGGCTAACACA--GTCAGAAATCGAAACGTACTCTCAACAGTTCGCTTAGGCAT   1212
                *   *  *** * *  *   * **   * **  ***  *      * ****     *

B73_3           AGAATTGGTAAAGAGAGTCG--TGTAAAATATCGAGTTCGCACATCTTGTTGTCTGATTA   1246
B4387_1         AGAATTGGTAAAGAGAGTCG--TGTAAAATATCGAGTTCGCACATTTTGTTGTCTGATTA   1246
B4387_2         AGAATTGGTAAAGAGAGTCG--TGTAAAATATCGAGTTCGCACATTTTGTTGTCTGATTA   1246
A287_1          GGAAGTTTTGCGGCATTCTGGCTACACAATAACAAGGGAAGACTCACTCGTGGCTGCAAC   1273
B73_1           GGAAGTTTTGCGGCATTCTGGCTACACAATAACAAGGGAAGACTCACTCGTGGCTGCAAC   1273
B73_2           GGAAGTTTTGCGGCATTCTGGCTACACAATAACAAGGGAAGACTCACTCGTGGCTGCAAC   1273
B4387_3         GGAAGTTTTGCGGCATTCTGGCTACACAATAACAAGGGAAGACTTACTCGTGGCTGCAAC   1272
                 *** *  *   *      *  *  * **** * **     **    *  ** ***

B73_3           TTGATTTTTCGCGAAACCATTTGATCATATGACAAGATGTGTATCCACCTTAACTTAATG   1306
B4387_1         TTGATTTTTGGCGAAACCATTTGATCATATGACAAGATGTGTATCTACCTTAACTTAATG   1306
B4387_2         TTGATTTTTGGCGAAACCATTTGATCATATGACAAGATGTGTATCTACCTTAACTTAATG   1306
A287_1          CC--TGCTTACTCAA----------------------AATCTATTCACA---CATGGTTA   1306
B73_1           CC--TGCTTACTCAA----------------------AATCTATTCACA---CATGGTTA   1306
B73_2           CC--TGCTTACTCAA----------------------AATCTATTCACA---CATGGTTA   1306
B4387_3         CC--TACTAGCTCAA----------------------AATTTATTCACA---CATGGTTA   1305
                    *  *     **                        * ***  **      *   *

B73_3           ATTTTTACCAAAATCATTAGGGGATTCATCAG      1338
B4387_1         ATTTTGATAAAAATCATTAGGGGATTCATCAG      1338
B4387_2         ATTTTGATAAAAATCATTAGGGGATTCATCAG      1338
A287_1          CGTTTTGGGGAAATTATGAGGGGATCTCTCAG      1338
B73_1           CGTTTTGGGGAAATTATGAGGGGATCTCTCAG      1338
B73_2           CGTTTTGGGGAAATTATGAGGGGATCTCTCAG      1338
B4387_3         CGCTTTGGGGAAATTATGAGGGGATCTCTCAG      1337
                   **     **** ** *******   ****
```

Table S2. Bacterial strains

Strain	Relevant phenotype or genotype*	Source/Reference
NEB5a		New England Biolab
MIT A2	*pks*$^+$	Mouse isolate, from the James Fox lab at MIT
MIT A21	*pks*$^+$	Mouse isolate, from the James Fox lab at MIT
MIT guinea pig isolate	*pks*$^+$	Guinea pig isolate, from the James Fox lab at MIT
NC101	*pks*$^+$	JANELLE C. ARTHUR, et al., 2012
NC101^{CmR}	*pks*$^+$ with a Cm resistance gene	This study
NC101 Δ*pks*	*pks* knockout	JANELLE C. ARTHUR, et al., 2012
DH10B pBAC	pBAC vector containing the *pks* island	Jean-Philippe Nougayrède, et al., 2021
DH10B pBAC-*pks*	Empty pBAC vector	Jean-Philippe Nougayrède, et al., 2021
EC100Dpir$^+$	F– mcrA Δ(mrr-hsdRMS-mcrBC) φ80dlacZΔM15 ΔlacX74 recA1 endA1 araD139 Δ(ara, leu)7697 galU galK λ– rpsL nupG pir+ (DHFR)	#ECP09500 (Lucigen)

MG1655^{NxR}	K-12 F– λ– ilvG– rfb-50 rph-1 Nxr	(Carraro et al., 2014)
E. coli Nissle 1917 strepR	*pks*$^{+}$ and Streptomycin resistance	Jean-Philippe Nougayrède, et al., 2021
eB-TP114	Evolved by accelerated laboratory evolution for 5 cycles in broth	This study
KN01	Smr, Spr Nissle 1917	Neil, et al., 2020

Table S3: Replicative and conjugative plasmids

Plasmid	Relevant phenotype or genotype*	Source/Reference
pBXB1	oriVpMB1, bxb1 integrase, bla (Apr)	Genbank: MK756311 (Neil et al., 2019)
pE-FLP	oriVpSC101ts, flp, Apr	Addgene #45978
pGRG25: CmR	Tn7 machinery to insert the aad7 and cat genes in the glmS terminator region	This study
pKill1	oriVpSC101ts, attPbxb1, FRT, 1 gRNA vs cat, aph-IIIa (Kmr), cas9	Genbank: MK756312 (Neil et al., 2019)
pNative-Cas9	Adapted from pKill1	This study
pNative-dCas9	oriVpSC101ts, attPbxb1, FRT, 1 gRNA vs cat, aph-IIIa (Kmr), dcas9	This study
pBAD-dCas9	oriVpSC101ts, attPbxb1, FRT, 1 gRNA vs cat, aph-IIIa (Kmr), dcas9, pBAD proter	This study
eB-TP114::tetB	eB-TP114Δaph-III::tetB	(Neil et al., 2021)
eB-TP114::pNative-Cas9	eB-TP114::tetB with inserted wild-type Cas9 with the original native promoter after bxb1- and flp-mediated deletion of tetB and oriVpSC101ts	This study
eB-TP114::pNative-dCas9	eB-TP114::tetB with inserted catalytically inactive Cas9 (dCas9) and the original native promoter after bxb1- and flp-mediated deletion of tetB and oriVpSC101ts	This study
eB-TP114::pBAD-dCas9	eB-TP114::tetB with dCas9 and the arabinose inducible promoter, pBAD, after bxb1- and flp-mediated deletion of tetB and oriVpSC101ts	This study

Table S4: Cloning, qPCR and sequencing primers

Capitalized letters in the sgRNA primers are the 20-nt spacer.

**Name**	**Sequence**	**Descriptions**
clbP_F	tatcatctcctgtgctgtatgctg	qPCR
clbP_R	cctgcatccgttggtgaatt	qPCR
Universal 16S_F	ggtgaatacgttcccgg	qPCR
Universal 16S_R	tacggctaccttgttacgactt	qPCR
irp1_F1	cactttctccgctaaggcca	qPCR
irp1_R1	gcatcattcacgatccgcac	qPCR
irp1_F2	agcacgttgtccatccagta	qPCR
irp1_R2	cggcgaaccctgctatgtat	qPCR
irp1_F3	aacagggtttcctcaccgtc	qPCR
irp1_R3	ttgccagggctaaccttcag	qPCR
clbA_287_fwd	GGCTACTGTATCTATAGTATgttttagagctagaaatagcaagt	sgRNA primers
clbA_287_rev	ATACTATAGATACAGTAGCCgaatctattatacagaaaaattttcctga	sgRNA primers
clbB_73_fwd	AATAAAAACCCCTGTTGACTgttttagagctagaaatagcaagt	sgRNA primers
clbB_73_rev	AGTCAACAGGGGTTTTTATTgaatctattatacagaaaaattttcctga	sgRNA primers
clbB_1667_fwd	TGGCAACCACGGTGTTTACCgttttagagctagaaatagcaagt	sgRNA primers
clbB_1667_rev	GGTAAACACCGTGGTTGCCAgaatctattatacagaaaaattttcctga	sgRNA primers
clbB_5400_fwd	TGTGCAAAATCGTGCAGCATgttttagagctagaaatagcaagt	sgRNA primers
clbB_5400_rev	ATGCTGCACGATTTTGCACAgaatctattatacagaaaaattttcctga	sgRNA primers
clbB_3673_fwd	GGAATGGATATTCAGGATACgttttagagctagaaatagcaagt	sgRNA primers
clbB_3673_rev	GTATCCTGAATATCCATTCCgaatctattatacagaaaaattttcctga	sgRNA primers
clbB_4387_fwd	GAACGCGATAGATCTATAGCgttttagagctagaaatagcaagt	sgRNA primers
clbB_4387_rev	GCTATAGATCTATCGCGTTCgaatctattatacagaaaaattttcctga	sgRNA primers
clbB_5610_fwd	AAGGTAGAGAGCGTATTACCgttttagagctagaaatagcaagt	sgRNA primers
clbB_5610_rev	GGTAATACGCTCTCTACCTTgaatctattatacagaaaaattttcctga	sgRNA primers
clbB_4129_fwd	GATACTGTCGCTATCAAAACgttttagagctagaaatagcaagt	sgRNA primers
clbB_4129_rev	GTTTTGATAGCGACAGTATCgaatctattatacagaaaaattttcctga	sgRNA primers

clbC_516_fwd	CTGGTGTAGAGACTATACACgttttagagctagaaatagcaagt	sgRNA primers
clbC_516_rev	GTGTATAGTCTCTACACCAGgaatctattatacagaaaaattttcc tga	sgRNA primers
clbC_2085_fwd	TCAACGTCTTCTCTTATTTCgttttagagctagaaatagcaagt	sgRNA primers
clbC_2085_rev	GAAATAAGAGAAGACGTTGAgaatctattatacagaaaaattttc ctga	sgRNA primers
clbC_2313_fwd	ACGAAAGGTACGCTTAACACgttttagagctagaaatagcaagt	sgRNA primers
clbC_2313_rev	GTGTTAAGCGTACCTTTCGTgaatctattatacagaaaaattttcct ga	sgRNA primers
clbC_1481_fwd	CGTTTTCCACTTGTATCACTgttttagagctagaaatagcaagt	sgRNA primers
clbC_1481_rev	AGTGATACAAGTGGAAAACGgaatctattatacagaaaaattttc ctga	sgRNA primers
clbH_1167_fwd	CCAGTTTTATACAGCCGCTCgttttagagctagaaatagcaagt	sgRNA primers
clbH_1167_rev	GAGCGGCTGTATAAAACTGGgaatctattatacagaaaaattttc ctga	sgRNA primers
clbH_1297_fwd	CCCCGGGAAACGCATCGCATgttttagagctagaaatagcaagt	sgRNA primers
clbH_1297_rev	ATGCGATGCGTTTCCCGGGGgaatctattatacagaaaaattttcc tga	sgRNA primers
clbI_331_fwd	ACCCACATCCCCCGGATAATgttttagagctagaaatagcaagt	sgRNA primers
clbI_331_rev	ATTATCCGGGGGATGTGGGTgaatctattatacagaaaaattttc ctga	sgRNA primers
clbI_170_fwd	CTTTGGCCTTGACGTAATCTgttttagagctagaaatagcaagt	sgRNA primers
clbI_170_rev	AGATTACGTCAAGGCCAAAGgaatctattatacagaaaaattttc ctga	sgRNA primers
clbJ_5578_fwd	GCCCCGACCGTAGGGAATACgttttagagctagaaatagcaagt	sgRNA primers
clbJ_5578_rev	GTATTCCCTACGGTCGGGGCgaatctattatacagaaaaattttcc tga	sgRNA primers
clbJ_3688_fwd	CGTGCGCATACTCAGAGCATgttttagagctagaaatagcaagt	sgRNA primers
clbJ_3688_rev	ATGCTCTGAGTATGCGCACGgaatctattatacagaaaaattttcc tga	sgRNA primers
clbL_412_fwd	TAATAGCTCGTTATCCCCTCgttttagagctagaaatagcaagt	sgRNA primers
clbL_412_rev	GAGGGGATAACGAGCTATTAgaatctattatacagaaaaattttc ctga	sgRNA primers
clbL_654_fwd	CTGTTGATCTATCTCATGGTgttttagagctagaaatagcaagt	sgRNA primers
clbL_654_rev	ACCATGAGATAGATCAACAGgaatctattatacagaaaaattttc ctga	sgRNA primers
clbL_993_fwd	CACCGCCACCGCCGCTCCTCgttttagagctagaaatagcaagt	sgRNA primers
clbL_993_rev	GAGGAGCGGCGGTGGCGGTGgaatctattatacagaaaaattttc ctga	sgRNA primers
clbN_759_fwd	GCGCCAGCTTTCGTGTGTGGgttttagagctagaaatagcaagt	sgRNA primers
clbN_759_rev	CCACACACGAAAGCTGGCGCgaatctattatacagaaaaattttc ctga	sgRNA primers
irp1_4019_fwd	AACCTTCCCTAGCCACTCCGgttttagagctagaaatagcaag t	sgRNA primers
irp1_4019_rev	CGGAGTGGCTAGGGAAGGTTgaatctattatacagaaaaatttt cctga	sgRNA primers

irp1_4129_fwd	GGCGCAGGAAGAGGCTCGGCgttttagagctagaaatagcaagt	sgRNA primers
irp1_4129_rev	GCCGAGCCTCTTCCTGCGCCgaatctattatacagaaaaattttcctga	sgRNA primers
irp1_397_fwd	GTCTTATGGGGGACGGCGCCgttttagagctagaaatagcaagt	sgRNA primers
irp1_397_rev	GGCGCCGTCCCCCATAAGACgaatctattatacagaaaaattttcctga	sgRNA primers
irp1_1595_fwd	CCCCGATTTCTCACCGGCCCgttttagagctagaaatagcaagt	sgRNA primers
irp1_1595_rev	GGGCCGGTGAGAAATCGGGGgaatctattatacagaaaaattttcctga	sgRNA primers
irp1_ko_fwd	aacgtgttctccggttgcgttgaggttgcgcaaagccagcgggtaatgtgatgggaattagccatggtcc	irp1 knockout
irp1_ko_rev	aacgcatggcgttccattgactttatgaaccttaggaaatgggaccgattgtgtaggctggagctgcttc	iIrp1 knockout
BACT1369F	cggtgaatacgttcycgg	qPCR
PROK1541R	aaggaggtgatccrgccgca	qPCR
clbB_F1	acacccgctgcgttagagttgc	qPCR
clbB_R1	ccgatggtcaaaggctgtgcgt	qPCR
clbC_F1	gctggaaatatcgccggtcggg	qPCR
clbC_R1	ttgcgcggattggggttttcca	qPCR
1492R	tacggytaccttgttacgactt	16S whole sequence
27F	agagtttgatcmtggctcag	16S whole sequence
aph-III upstream TP114	ctatgccacgctacgccgctac	sequencing
aph-III downstream TP114	ctggtcacggcggcgaatatcc	sequencing
ClbA upstream	tcacgctatacacattgctaacag	*pks*$^{+}$ detection
ClbA downstream	tgtgaatggcacgattatgcggg	*pks*$^{+}$ detection
pkill1	gctcaacagtcacacatagacagcc	sequencing

Cm spacer in original pKill1	taacacgccacatcttgcga	sequencing
KO of clbP verification_dwn upstream	cgaatacggagcgacctcgatgc	*pks*+ detection
KO of clbP verification_dwn	gtcagcgacggcatccaccatc	*pks*+ detection
sgclbB_4387	gctatagatctatcgcgttc	sgRNA detection
sgclbC_2313	gtgttaagcgtacctttcgt	sgRNA detection
PstI_backbone	ggtgccctgaatgaactgca	Mutation of Cas9 to dCas9
D10A	tgtgccgatagctaagcctat	Mutation of Cas9 to dCas9
D10A	ataggcttagctatcggcaca	Mutation of Cas9 to dCas9
H840A	tggaacaatggcatcgacatc	Mutation of Cas9 to dCas9
H840A	gatgtcgatgccattgttcca	Mutation of Cas9 to dCas9
BamHI_backbone	actatcaaaaccaccatatttttttg	Mutation of Cas9 to dCas9
pBAD_Promoter_fwd	accccagcttcaaaagcgctatcgatgcataatgtgcc	Construction of pBAD_dCas9
pBAD_Promoter_rev	attgagtatttcttatccatggtatatctccttattaaagttaaac	Construction of pBAD_dCas9
pKILL1-cas9_Backbone_fwd	atggataagaaatactcaataggcttag	Construction of pBAD_dCas9
pKILL1-cas9_Backbone_rev	agcgcttttgaagctggg	Construction of pBAD_dCas9

Table S5. Promoter and coding DNA sequences

Name	Length	sequences
pNative promoter	240 bp	tttatcagccataaaacaatacttaatactatagaatgataacaaaataaactactttttaaaagaattttgtgttata<br>atctatttattattaagtattgggtaatatttttgaagagatattttgaaaaagaaaaattaaagcatattaaactaat<br>ttcggaggtcattaaaactattattgaaatcatcaaactcattatggatttaatttaaactttttattttaggaggcaa<br>aa
pBAD promoter	1346 bp	atcgatgcataatgtgcctgtcaaatggacgaagcagggattctgcaaaccctatgctactccgtcaagccgt<br>caattgtctgattcgttaccaattatgacaacttgacggctacatcattcactttttcttcacaaccggcacggaac<br>tcgctcgggctggccccggtgcattttttaaatacccgcgagaaatagagttgatcgtcaaaaccaacattgcg<br>accgacggtggcgataggcatccgggtggtgctcaaaagcagcttcgcctggctgatacgttggtcctcgcg<br>ccagcttaagacgctaatccctaactgctggcggaaaagatgtgacagacgcgacggcgacaagcaaacat<br>gctgtgcgacgctggcgatatcaaaattgctgtctgccaggtgatcgctgatgtactgacaagcctcgcgtac<br>ccgattatccatcggtggatggagcgactcgttaatcgcttccatgcgccgcagtaacaattgctcaagcagat<br>ttatcgccagcagctccgaatagcgcccttccccttgcccggcgttaatgatttgcccaaacaggtcgctgaaa<br>tgcggctggtgcgcttcatccgggcgaaagaaccccgtattggcaaatattgacggccagttaagccattcat<br>gccagtaggcgcgcggacgaaagtaaacccactggtgataccattcgcgagcctccggatgacgaccgta<br>gtgatgaatctctcctggcgggaacagcaaaatatcacccggtcggcaaacaaattctcgtccctgatttttca<br>ccaccccctgaccgcgaatggtgagattgagaatataacctttcattcccagcggtcggtcgataaaaaaatc<br>gagataaccgttggcctcaatcggcgttaaacccgccaccagatgggcattaaacgagtatcccggcagca<br>ggggatcattttgcgcttcagccatacttttcatactcccgccattcagagaagaaaccaattgtccatattgcat<br>cagacattgccgtcactgcgtcttttactggctcttctcgctaaccaaaccggtaaccccgcttattaaaagcatt<br>ctgtaacaaagcgggaccaaagccatgacaaaaacgcgtaacaaaagtgtctataatcacggcagaaaagt<br>ccacattgattatttgcacggcgtcacactttgctatgccatagcatttttatccataagattagcggatcctacct<br>gacgctttttatcgcaactctctactgtttctccatacccgtttttttgggctagccctgtagaaataattgtttaactt<br>taataaggagatatacc
Cas9	4107 bp	atggataagaaatactcaataggcttagatatcggcacaaatagcgtcggatgggcggtgatcactgatgaat<br>ataaggttccgtctaaaaagttcaaggttctgggaaatacagaccgccacagtatcaaaaaaaatcttataggg<br>gctcttttatttgacagtggagagacagcggaagcgactcgtctcaaacggacagctcgtagaaggtatacac<br>gtcggaagaatcgtatttgttatctacaggagatttttcaaatgagatggcgaaagtagatgatagtttctttcat<br>cgacttgaagagtcttttttggtggaagaagacaagaagcatgaacgtcatcctatttttggaaatatagtagat<br>gaagttgcttatcatgagaaatatccaactatctatcatctgcgaaaaaaattggtagattctactgataaagcgg<br>atttgcgcttaatctatttggccttagcgcatatgattaagtttcgtggtcatttttttgattgagggagatttaaatcct<br>gataatagtgatgtggacaaactatttatccagttggtacaaacctacaatcaattatttgaagaaaaccctatta<br>acgcaagtggagtagatgctaaagcgattctttctgcacgattgagtaaatcaagacgattagaaaatctcattg<br>ctcagctccccggtgagaagaaaaatggcttatttgggaatctcattgctttgtcattgggtttgacccctaatttt<br>aaatcaaattttgatttggcagaagatgctaaattacagctttcaaaagatacttacgatgatgatttagataattta<br>ttggcgcaaattggagatcaatatgctgatttgtttttggcagctaagaatttatcagatgctattttactttcagata<br>tcctaagagtaaatactgaaataactaaggctcccctatcagcttcaatgattaaacgctacgatgaacatcatc<br>aagacttgactcttttaaaagctttagttcgacaacaacttccagaaaagtataaagaaatcttttttgatcaatca<br>aaaaacggatatgcaggttatattgatgggggagctagccaagaagaattttataaatttatcaaaccaattttta<br>gaaaaaatggatggtactgaggaattattggtgaaactaaatcgtgaagatttgctgcgcaagcaacggacct<br>ttgacaacggctctattccccatcaaattcacttgggtgagctgcatgctattttgagaagacaagaagactttta<br>tccatttttaaaagacaatcgtgagaagattgaaaaaatcttgacttttcgaattccttattatgttggtccattggc<br>gcgtggcaatagtcgttttgcatggatgactcggaagtctgaagaaacaattaccccatggaattttgaagaag<br>ttgtcgataaaggtgcttcagctcaatcatttattgaacgcatgacaaactttgataaaaatcttccaaatgaaaa<br>agtactaccaaaacatagtttgctttatgagtattttacggtttataacgaattgacaaaggtcaaatatgttactga<br>aggaatgcgaaaaccagcatttctttcaggtgaacagaagaaagccattgttgatttactcttcaaaacaaatcg<br>aaaagtaaccgttaagcaattaaaagaagattatttcaaaaaaatagaatgttttgatagtgttgaaatttcagga

		gttgaagatagatttaatgcttcattaggtacctaccatgatttgctaaaaattattaaagataaagatttttttggata<br>atgaagaaaatgaagatatcttagaggatattgttttaacattgaccttatttgaagatagggagatgattgagga<br>aagacttaaaacatatgctcacctctttgatgataaggtgatgaaacagcttaaacgtcgccgttatactggttg<br>gggacgtttgtctcgaaaattgattaatggtattagggataagcaatctggcaaaacaatattagatttttttgaaat<br>cagatggttttgccaatcgcaattttatgcagctgatccatgatgatagtttgacatttaaagaagacattcaaaa<br>agcacaagtgtctggacaaggcgatagtttacatgaacatattgcaaatttagctggtagccctgctattaaaaa<br>aggtattttacagactgtaaaagttgttgatgaattggtcaaagtaatggggcggcataagccagaaaatatcgt<br>tattgaaatggcacgtgaaaatcagacaactcaaaagggccagaaaaattcgcgagagcgtatgaaacgaat<br>cgaagaaggtatcaaagaattaggaagtcagattcttaaagagcatcctgttgaaaatactcaattgcaaaatg<br>aaaagctctatctctattatctccaaaatggaagagacatgtatgtggaccaagaattagatattaatcgtttaagt<br>gattatgatgtcgatcacattgttccacaaagtttccttaaagacgattcaatagacaataaggtcttaacgcgttc<br>tgataaaaatcgtggtaaatcggataacgttccaagtgaagaagtagtcaaaaagatgaaaaactattggaga<br>caacttctaaacgccaagttaatcactcaacgtaagtttgataatttaacgaaagctgaacgtggaggtttgagt<br>gaacttgataaagctggttttatcaaacgccaattggttgaaactcgccaaatcactaagcatgtggcacaaatt<br>ttggatagtcgcatgaatactaaatacgatgaaaatgataaacttattcgagaggttaaagtgattaccttaaaat<br>ctaaattagtttctgacttccgaaaagatttccaattctataaagtacgtgagattaacaattaccatcatgcccat<br>gatgcgtatctaaatgccgtcgttggaactgctttgattaagaaatatccaaaacttgaatcggagtttgtctatg<br>gtgattataaagtttatgatgttcgtaaaatgattgctaagtctgagcaagaaataggcaaagcaaccgcaaaat<br>atttcttttactctaatatcatgaacttcttcaaaacagaaattacacttgcaaatggagagattcgcaaacgccct<br>ctaatcgaaactaatggggaaactggagaaattgtctgggataaagggcgagattttgccacagtgcgcaaa<br>gtattgtccatgccccaagtcaatattgtcaagaaaacagaagtacagacaggcggattctccaaggagtcaa<br>ttttaccaaaaagaaattcggacaagcttattgctcgtaaaaaagactgggatccaaaaaaatatggtggttttg<br>atagtccaacggtagcttattcagtcctagtggttgctaaggtggaaaaagggaaatcgaagaagttaaaatcc<br>gttaaagagttactagggatcacaattatggaaagaagttcctttgaaaaaaatccgattgactttttagaagcta<br>aaggatataaggaagttaaaaaagacttaatcattaaactacctaaatatagtctttttgagttagaaaacggtcg<br>taaacggatgctggctagtgccggagaattacaaaaaggaaatgagctggctctgccaagcaaatatgtgaa<br>tttttatatttagctagtcattatgaaaagttgaagggtagtccagaagataacgaacaaaaacaattgtttgtgg<br>agcagcataagcattatttagatgagattattgagcaaatcagtgaattttctaagcgtgttattttagcagatgcc<br>aatttagataaagttcttagtgcatataacaaacatagagacaaaccaatacgtgaacaagcagaaaatattatt<br>catttatttacgttgacgaatcttggagctcccgctgcttttaaatattttgatacaacaattgatcgtaaacgatata<br>cgtctacaaaagaagttttagatgccactcttatccatcaatccatcactggtctttatgaaacacgcattgatttg<br>agtcagctaggaggtgactga
dCas9	4107 bp	atggataagaaatactcaataggcttaGCTatcggcacaaatagcgtcggatgggcggtgatcactgatga<br>atataaggttccgtctaaaaagttcaaggttctgggaaatacagaccgccacagtatcaaaaaaaatcttatag<br>gggctcttttatttgacagtggagagacagcggaagcgactcgtctcaaacggacagctcgtagaaggtata<br>cacgtcggaagaatcgtatttgttatctacaggagattttttcaaatgagatggcgaaagtagatgatagtttcttt<br>catcgacttgaagagtcttttttggtggaagaagacaagaagcatgaacgtcatcctatttttggaaatatagtag<br>atgaagttgcttatcatgagaaatatccaactatctatcatctgcgaaaaaaattggtagattctactgataaagc<br>ggatttgcgcttaatctatttggccttagcgcatatgattaagtttcgtggtcatttttttgattgagggagatttaaat<br>cctgataatagtgatgtggacaaactatttatccagttggtacaaacctacaatcaattatttgaagaaaaccctа<br>ttaacgcaagtggagtagatgctaaagcgattctttctgcacgattgagtaaatcaagacgattagaaaatctca<br>ttgctcagctccccggtgagaagaaaaatggcttatttgggaatctcattgctttgtcattgggtttgacccctaat<br>tttaaatcaaattttgatttggcagaagatgctaaattacagctttcaaaagatacttacgatgatgatttagataatt<br>tattggcgcaaattggagatcaatatgctgatttgtttttggcagctaagaatttatcagatgctattttactttcaga<br>tatcctaagagtaaatactgaaataactaaggctcccctatcagcttcaatgattaaacgctacgatgaacatca<br>tcaagacttgactcttttaaaagctttagttcgacaacaacttccagaaaagtataaagaaatctttttttgatcaatc<br>aaaaaacggatatgcaggttatattgatgggggagctagccaagaagaattttataaatttatcaaaccaattta<br>gaaaaaatggatggtactgaggaattattggtgaaactaaatcgtgaagatttgctgcgcaagcaacggacct<br>ttgacaacggctctattccccatcaaattcacttgggtgagctgcatgctattttgagaagacaagaagactttta<br>tccatttttaaaagacaatcgtgagaagattgaaaaaatcttgacttttcgaattccttattatgttggtccattggc<br>gcgtggcaatagtcgttttgcatggatgactcggaagtctgaagaaacaattaccccatggaattttgaagaag

		ttgtcgataaaggtgcttcagctcaatcatttattgaacgcatgacaaactttgataaaaatcttccaaatgaaaa<br>agtactaccaaaacatagtttgctttatgagtattttacggtttataacgaattgacaaaggtcaaatatgttactga<br>aggaatgcgaaaaccagcatttctttcaggtgaacagaagaaagccattgttgatttactcttcaaaacaaatcg<br>aaaagtaaccgttaagcaattaaaagaagattatttcaaaaaaatagaatgttttgatagtgttgaaatttcagga<br>gttgaagatagatttaatgcttcattaggtacctaccatgatttgctaaaaattattaaagataaagatttttttggata<br>atgaagaaaatgaagatatcttagaggatattgttttaacattgaccttatttgaagatagggagatgattgagga<br>aagacttaaaacatatgctcacctctttgatgataaggtgatgaaacagcttaaacgtcgccgttatactggttg<br>gggacgtttgtctcgaaaattgattaatggtattagggataagcaatctggcaaaacaatattagatttttttgaaat<br>cagatggttttgccaatcgcaattttatgcagctgatccatgatgatagtttgacatttaaagaagacattcaaaa<br>agcacaagtgtctggacaaggcgatagtttacatgaacatattgcaaatttagctggtagccctgctattaaaaa<br>aggtattttacagactgtaaaagttgttgatgaattggtcaaagtaatggggcggcataagccagaaaatatcgt<br>tattgaaatggcacgtgaaaatcagacaactcaaaagggccagaaaaattcgcgagagcgtatgaaacgaat<br>cgaagaaggtatcaaagaattaggaagtcagattcttaaagagcatcctgttgaaaatactcaattgcaaaatg<br>aaaagctctatctctattatctccaaaatggaagagacatgtatgtggaccaagaattagatattaatcgtttaagt<br>gattatgatgtcgatGCCattgttccacaaagtttccttaaagacgattcaatagacaataaggtcttaacgcgt<br>tctgataaaaatcgtggtaaatcggataacgttccaagtgaagaagtagtcaaaaagatgaaaaactattgga<br>gacaacttctaaacgccaagttaatcactcaacgtaagtttgataatttaacgaaagctgaacgtggaggtttga<br>gtgaacttgataaagctggttttatcaaacgccaattggttgaaactcgccaaatcactaagcatgtggcacaa<br>attttggatagtcgcatgaatactaaatacgatgaaaatgataaacttattcgagaggttaaagtgattaccttaa<br>aatctaaattagtttctgacttccgaaaagatttccaattctataaagtacgtgagattaacaattaccatcatgcc<br>catgatgcgtatctaaatgccgtcgttggaactgctttgattaagaaatatccaaaacttgaatcggagtttgtcta<br>tggtgattataaagtttatgatgttcgtaaaatgattgctaagtctgagcaagaaataggcaaagcaaccgcaaa<br>atatttcttttactctaatatcatgaacttcttcaaaacagaaattacacttgcaaatggagagattcgcaaacgcc<br>ctctaatcgaaactaatggggaaactggagaaattgtctgggataaagggcgagattttgccacagtgcgcaa<br>agtattgtccatgccccaagtcaatattgtcaagaaaacagaagtacagacaggcggattctccaaggagtca<br>attttaccaaaaagaaattcggacaagcttattgctcgtaaaaaagactgggatccaaaaaaatatggtggtttt<br>gatagtccaacggtagcttattcagtcctagtggttgctaaggtggaaaaagggaaatcgaagaagttaaaat<br>ccgttaaagagttactagggatcacaattatggaaagaagttcctttgaaaaaaatccgattgactttttagaagc<br>taaaggatataaggaagttaaaaaagacttaatcattaaactacctaaatatagtctttttgagttagaaaacggt<br>cgtaaacggatgctggctagtgccggagaattacaaaaaggaaatgagctggctctgccaagcaaatatgtg<br>aatttttatatttagctagtcattatgaaaagttgaagggtagtccagaagataacgaacaaaaacaattgtttgt<br>ggagcagcataagcattatttagatgagattattgagcaaatcagtgaattttctaagcgtgttattttagcagatg<br>ccaatttagataaagttcttagtgcatataacaaacatagagacaaaccaatacgtgaacaagcagaaaatatt<br>attcatttatttacgttgacgaatcttggagctcccgctgcttttaaatattttgatacaacaattgatcgtaaacgat<br>atacgtctacaaaagaagttttagatgccactcttatccatcaatccatcactggtctttatgaaacacgcattgat<br>ttgagtcagctaggaggtgactga

Supplementary Files

This is a list of supplementary files associated with this preprint. Click to download.

- nreditorialpolicychecklist10012025.pdf
- nrreportingsummary10012025.pdf
